# Living Donor Kidney Transplantation in Patients With Donor-Specific HLA Antibodies After Desensitization With Immunoadsorption

**DOI:** 10.3389/fmed.2021.781491

**Published:** 2021-12-17

**Authors:** Florian Kälble, Caner Süsal, Luiza Pego da Silva, Claudius Speer, Louise Benning, Christian Nusshag, Lien Pham, Hien Tran, Matthias Schaier, Claudia Sommerer, Jörg Beimler, Arianeb Mehrabi, Martin Zeier, Christian Morath

**Affiliations:** ^1^Department of Nephrology, University Hospital Heidelberg, Heidelberg, Germany; ^2^Transplant Immunology Research Center of Excellence, Koç University Hospital, Istanbul, Turkey; ^3^Transplantation-Immunology, Institute of Immunology, University Hospital Heidelberg, Heidelberg, Germany; ^4^Department of General, Visceral and Transplantation Surgery, University Hospital Heidelberg, Heidelberg, Germany

**Keywords:** desensitization, immunoadsorption, donor-specific antibody (DSA), antibody-mediated rejection (AMR), kidney transplantation

## Abstract

Due to the current organ shortage, living donor kidney transplantation is increasingly performed across HLA (human leukocyte antigen) or ABO antibody barriers. There is still uncertainty about the risk of antibody-mediated rejection (AMR) episodes, which may limit long-term graft survival. From March 2007 to December 2016, 58 sensitized living donor kidney transplant candidates were identified and 38 patients eventually included in the study: 36 patients (95%) had pre-transplant and pre-desensitization Luminex-detected donor-specific HLA antibodies (DSA), and 17/36 patients (47%) in addition had a positive crossmatch result. Two patients had no detectable DSA but a positive CDC B-cell crossmatch result. Patients were treated with pre- and post-transplant apheresis and powerful immunosuppression including the anti-CD20 antibody rituximab (*N* = 36) in combination with thymoglobulin (*N* = 20) or anti-IL2 receptor antibody (*N* = 18). The results of the 38 successfully desensitized and transplanted patients were retrospectively compared to the results of 76 matched standard-risk recipients. Desensitized patients showed patient and graft survival rates similar to that of standard-risk recipients (*P* = 0.55 and *P* = 0.16, respectively). There was a trend toward reduced death-censored graft survival in desensitized patients (*P* = 0.053) which, however, disappeared when the 34 patients who were transplanted after introduction of sensitive Luminex testing were analyzed (*P* = 0.43). The incidence of rejection episodes without borderline changes were in desensitized patients with 21% similar to the 18% in standard-risk patients (*P* = 0.74). Thirty-six patients had pre-transplant HLA class I and/or II DSA that were reduced by 85 and 81%, respectively, during pre-transplant desensitization (*P* < 0.001 for both). On day 360 after transplantation, 20 of 36 (56%) patients had lost their DSA. The overall AMR rate was 6% in these patients, but as high as 60% in 5 (14%) patients with persistent and *de novo* DSA during year 1; 2 (40%) of whom lost their graft due to AMR. Eleven (31%) patients with persistent DSA but without *de novo* DSA had an AMR rate of 18% without graft loss while one patient lost her graft without signs of AMR. Our desensitization protocol for pre-sensitized living donor kidney transplant recipients with DSA resulted in good graft outcomes with side effects and rejection rates similar to that of standard-risk recipients. Adequate patient selection prior to transplantation and frequent immunological monitoring thereafter is critical to minimize rejection episodes and subsequent graft loss.

## Introduction

The increasing number of patients with chronic kidney disease and the ongoing organ shortage have led to efforts to increase the number of living donor transplants. One possibility is living donor kidney transplantation across the human leukocyte antigen (HLA) barrier after desensitization prior to transplantation. A study by Montgomery et al. showed that the overall survival rate of patients who were desensitized for living donor kidney transplantation was significantly higher than the survival rate of patients waiting for a compatible allograft from a deceased donor (1). These results were later confirmed in a larger multicenter study from the United States, but did not hold up when the same analysis was performed for patients transplanted in the United Kingdom (2, 3). Several protocols exist for desensitization of kidney transplant recipients, that are all based on rapid reduction of HLA antibodies before transplantation and strong immunosuppression to permanently suppress *de novo* HLA antibody formation thereafter. Immunoadsorption (IA) has been shown to be effective in rapidly removing HLA antibodies before transplantation. Bartel et al. published encouraging results from 68 HLA-sensitized deceased donor kidney transplant recipients desensitized by IA (4). We demonstrated that HLA antibody removal by IA was effective in 10 recipients of crossmatch-positive living donor kidney transplants (5). A larger analysis of 23 HLA-sensitized recipients from our center confirmed the excellent results with a graft and patient survival rate of 100% at two years and a low rate of treatment-related adverse events and rejection episodes (6).

Since 2006, we have been consistently using this desensitization protocol and have gained broader experience with desensitization in a total of 58 patients, 38 of whom were eventually included in this study. The aim of this study was to compare the results of these 38 successfully desensitized patients with the results of 76 matched standard-risk recipients. The primary outcome measures were graft and patient survival while the secondary outcome measures included effectiveness of antibody-removal by desensitization with immunoadsorption, graft function, biopsy-proven rejection episodes, complications, and the course of donor-specific HLA antibodies (DSA) after transplantation. In addition, we aimed to determine the impact of the introduction of the highly sensitive Luminex assay to our routine in 2009 on the outcomes of these HLA antibody-incompatible transplants.

## Materials and Methods

### “Heidelberg Algorithm” Criteria and Patient Selection

Patients transplanted under the “Heidelberg Algorithm” by December 2016 were considered for inclusion in the study. The “Heidelberg Algorithm” was developed in 2005 and applied since April 2006 to identify and treat patients on the Heidelberg waiting list who are at particularly high risk for AMR after transplantation (Supplementary Table 1) (7–9). Based on results from the *Collaborative Transplant Study*, patients were considered at increased risk if they had a CDC-PRA-DTT ≥ 85% (current or past), HLA class I and II antibody positivity in ELISA screening, or HLA class I antibody positivity in ELISA screening at re-transplantation (donor-independent criteria), or a positive CDC B-cell crossmatch in re-transplant recipients with HLA class II antibody positivity in ELISA screening, or a positive CDC T-cell crossmatch (donor-dependent criteria). In addition, recipients of living donor kidneys were considered as high-risk if they had a DSA ≥ 1,000 MFI (after April 2009) (7–9). These patients were treated and monitored according to a specific algorithm that, for living donor kidney transplant recipients, included pre- and post-transplant desensitization, powerful antibody induction therapy and immunosuppression and close post-transplant antibody monitoring together with protocol biopsies (Supplementary Table 1).

### Desensitization Therapy

IA was performed with Peptid-GAM-coated Globaffin columns that specifically bind IgG1, 2, and 4 and intermediately strong IgG3 and weakly IgM antibodies (Fresenius Medical Care, Bad Homburg, Germany) on an ADAsorb device (medicap clinic GmbH, Ulrichstein, Germany) together with an AS.TEC 204 centrifuge (Fresenius Medical Care). Treatment was repeated before transplantation until the CDC- and ELISA-crossmatch results became negative. In addition, DSA had to be negative in ELISA screening and below 1,000 MFI in Luminex testing (from March 2009). IA was applied on alternate days. Anticoagulation during IA consisted of 1,500 units of heparin per hour together with sodium citrate (ACD-A, Fresenius Kabi AG, Bad Homburg, Germany) at an infusion rate of 1:23 (citration infusion: blood flow). In patients with risk of bleeding, especially after surgery, the treatments were conducted without heparin and a sodium citrate infusion rate of 1:16. Resulting hypocalcemia was treated with calcium gluconate (10%, B. Braun, Melsungen, Germany). In patients with proven or suspected DSA of the IgM isotype, plasmapheresis was used additionally to account for the reduced IgM removal efficacy of IA.

After transplantation, apheresis treatments were continued in all patients on alternate days until good allograft function was achieved with a serum creatinine of <2 mg/dL and the DSA remained negative in ELISA screening and below a cut-off of 1,000 MFI in Luminex single antigen testing (from March 2009).

### Immunosuppression and Infection Prophylaxis

In sensitized patients, immunosuppression with tacrolimus (target trough levels month 1: 10–15 μg/L, month 2: 10–12 μg/L, month 3: 8–10 μg/L, beyond year 1: 5–8 μg/L), enteric-coated mycophenolate sodium (720 mg twice daily) and methylprednisolone (20 mg during desensitization, 250 mg on day 0, tapered to 20 mg on day 9 after surgery) was started with the initiation of IA. Induction therapy was applied with thymoglobulin (*N* = 20, 2–7 infusions of 1.5 mg/kg body weight with a target lymphocyte count of ~0.2/nL within the first 14 days after transplantation), or basiliximab (*N* = 18, 20 mg on days 0 and 4 after transplantation). From May 2009, the induction therapy was changed from basiliximab to thymoglobulin in all sensitized patients due to an increased frequency of T cell-mediated rejection episodes. Basiliximab continued to be given in those patients who possessed low-level DSA that were identified in Luminex testing only but who had a negative crossmatch result (and a soluble CD30 (sCD30) concentration below 80 ng/mL, from October 2016).

In addition, rituximab was administered at a single dose of 375 mg/m^2^ body surface after the last IA treatment on day-1 before surgery (*N* = 36).

Standard risk patients received immunosuppression with cyclosporine A (target trough levels month 1: 180–200 μg/L, month 2: 150–180 μg/L, month 3–year 1: 120–150 μg/L, beyond year 1: 100–120 μg/L) together with enteric-coated mycophenolate sodium and methylprednisolone at the same dose as in sensitized recipients. Induction therapy was conducted with basiliximab on days 0 and 4 after transplantation.

Prophylaxis with valganciclovir was performed for 3 months in cytomegalovirus-positive patients and/or recipients of a cytomegalovirus-positive organ. Fungal prophylaxis consisted of 1 mL of nystatin four times daily for 3 months. Pneumocystis jirovecii prophylaxis was conducted by alternate day administration of trimethoprim (160 mg) and sulfamethoxazole (800 mg) for 6 months.

### Immunology

CDC crossmatches were performed with unseparated peripheral blood mononuclear cells as well as isolated donor T and B lymphocytes using the standard CDC technique without anti-human immunoglobulin enhancement. In addition, a solid-phase ELISA crossmatch assay (AbCross, Biotest, Dreieich, Germany) was used. PRA screenings were performed using CDC and ELISA techniques. DSA of the IgG isotype against HLA antigens were determined by ELISA and, since March 2009, in addition by Luminex technologies using the AbIdent kits of Biotest (Dreieich, Germany), and the LABScreen Single Antigen kit of One Lambda (Canoga Park, CA, USA), respectively. For the detection of DSA of the IgM isotype by Luminex, 1:100 diluted PE-conjugated F(ab')_2_ fragments of donkey anti-human IgM, Fc antibodies (Dianova, Hamburg, Germany) were used. HLA typings of donors and recipients were performed using PCR-SSP and sequencing. The HLA alloantibodies were measured after transplantation on days 7, 30, 180, 360, and every 6 months thereafter. Additional testing was performed if deterioration of allograft function was noted. We and others use different cut-offs for the determination of DSA before and after transplantation (10). While our pre-transplant cut-off is 1,000 MFI, post-transplant DSA are considered relevant at a cut-off of 500 MFI to capture low level *de novo* DSA and because *in vivo* antibody adsorption in the allograft may lead to falsely low antibody reactivity. Since October 2016, pre-transplant and pre-desensitization serum samples were further tested for sCD30 using human sCD30 Instant ELISA (eBioscience, San Diego, CA) according to the manufacturer's instructions. An sCD30 concentration ≥80 ng/mL before transplantation was used as cut-off for positivity.

### Statistical Analysis

The primary outcome measure was graft and patient survival while secondary outcome measures included effectiveness of antibody-removal by desensitization with immunoadsorption, graft function, biopsy-proven rejection episodes, complications, and the course of DSA after transplantation. In addition, the impact of the introduction of the highly sensitive Luminex assay to clinical routine in 2009 on the outcomes of HLA antibody-incompatible transplants was determined.

Data are given as median and range, mean and standard error of the mean, or number and percent. Statistical analyses were performed using SPSS Statistics 28 (IBM). For group comparisons *t*-test and Mann-Whitney-*U*-test were used, chi-square test for categorial variables.

Reduction of immunoglobulins or DSA was calculated with the Wilcoxon matched-pairs signed rank test. Graft survival was calculated according to the Kaplan-Meier method.

## Results

### Patient Selection and Baseline Demographics

**Figure 1:**
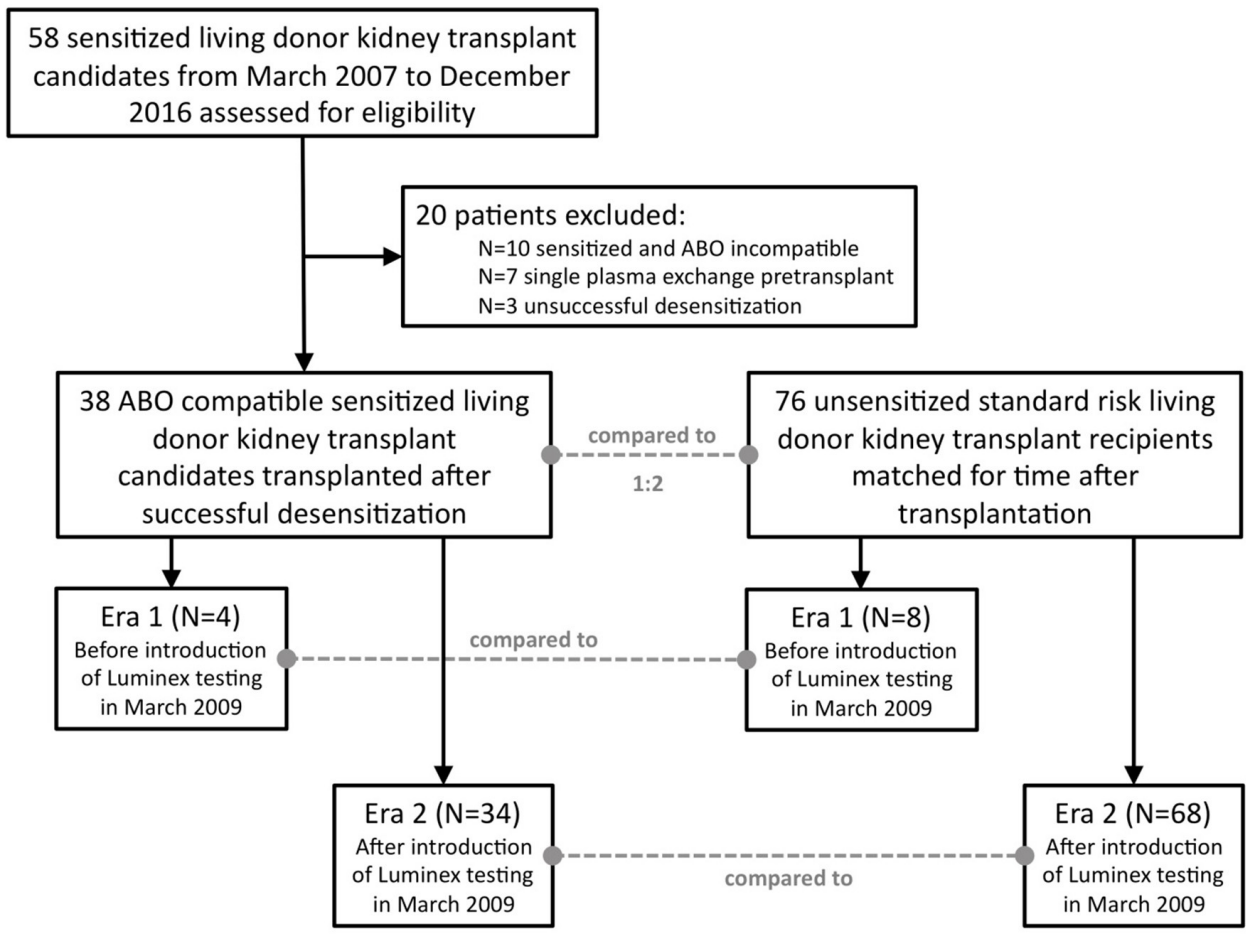
Flow chart for patient selection. Fifty-eight sensitized patients were screened, and 38 patients eventually included in the analysis. Each sensitized patient (*N* = 38) was matched with two standard risk recipients (*N* = 76) for time after transplantation, i.e., the first standard risk recipient transplanted before and the first transplanted after surgery of the desensitized candidate. Patients were further stratified according to the date of transplantation, either before March 2009 or thereafter. In March 2009, highly sensitive Luminex testing was introduced into clinical routine at our center.

Fifty-eight HLA-sensitized living donor kidney transplant candidates who met the “Heidelberg Algorithm” criteria and were desensitized between March 2007 and December 2016 were identified and screened for eligibility (Supplementary Table 1; Figure 1). Twenty patients were excluded from the analysis due to transplantation in the co-presence of a major ABO incompatibility (*N* = 10), a single pre-transplant plasmapheresis treatment only due to very low DSA levels (*N* = 7), or an unsuccessful desensitization (*N* = 3). The 38 successfully desensitized patients met at least one criterion of the “Heidelberg algorithm”: 36 patients (95%) had pre-transplant and pre-desensitization DSA, and 17/36 patients (47%) in addition had a positive crossmatch result. Two patients (number 22 and 23) had no detectable DSA but a positive CDC B-cell crossmatch result.

Results of 38 desensitized living donor kidney transplant recipients were analyzed and retrospectively compared to results of 76 standard-risk living donor kidney recipients matched for the time of transplantation. Patients were further divided into two different groups derived from two different eras, before (*N* = 4) and after (*N* = 34) the introduction of highly sensitive Luminex testing in March 2009, and analyzed separately.

Most demographic data were comparable between the desensitized and standard-risk recipients (Table 1). In addition to the degree of sensitization, significant differences between the groups were a higher number of previous transplants (*P* = 0.01), a lower number of related donors (*P* = 0.01), a longer postoperative hospital stay (18 vs. 13 days) presumably due to the more complex procedure (*P* < 0.001), and the pre- and post-operative treatment (*P* < 0.001) in desensitized patients. The majority of desensitized patients received immunosuppression with tacrolimus except for one patient who received cyclosporine A due to tacrolimus intolerance. In contrast, 72% of standard-risk recipients received cyclosporine A treatment according to center protocol for immunological low-risk recipients at that time.

**Table 1:** Baseline patient characteristics.

	**Standard risk (*N* = 76)**	**Desensitized** **(*N* = 38)**	***P* value**
**Recipient characteristics**			
Female sex, N (%)	26 (34)	18 (47)	0.22
Age, median (range)	39 (16–70)	44 (20–62)	0.34
Caucasian race, N (%)	75 (99)	38 (100)	1.0
Cause of ESRD, N (%)			0.62
Diabetes	3 (4)	0 (0)	
Hypertension	7 (9)	2 (5)	
Glomerulonephritis	35 (46)	14 (37)	
Pyelonephritis	7 (9)	3 (8)	
ADPKD	10 (13)	9 (24)	
Other	10 (13)	6 (16)	
Unknown	4 (5)	4 (11)	
Comorbidities^*^, N (%)			
Diabetes	3 (4)	7 (18)	0.015
Hypertension	57 (75)	27 (71)	0.66
Cardiovascular event	5 (7)	2 (5)	1.0
N of previous tx, N (0/1/2)	70/6/0	23/11/4	<0.001
Mode of pre-tx dialysis, N (%)			0.24
HD	48 (62)	30 (79)	
PD	8 (11)	3 (8)	
Preemptive tx	20 (26)	5 (13)	
Years on dialysis before last tx, median (range)	0.8 (0–17)	1 (0–9)	0.71
**Donor characteristics**			
Female sex, N (%)	44 (58)	21 (55)	0.84
Age, median (range)	50 (27–77)	50 (25–75)	0.67
Related donor, N (%)	51 (67)	15 (39)	0.008
**Pre-tx immunological parameters**			
CDC T-Cell PRA %, median (range)	0 (0–5)	0 (0–98)	<0.001
HLA-A+B+DR mismatches, N (%)			
0–1	12 (16)	3 (8)	0.38
2–4	48 (63)	30 (79)	0.13
5–6	16 (21)	5 (13)	0.44
CDC-XM result positive, N (%)	2 (3)^a^	19 (50)	<0.001
B-cell	1 (1)	12 (32)	<0.001
T-cell	0 (0)	1 (3)	0.33
U+B-cell	0 (0)	3 (8)	0.035
U+B+T-cell	1 (1)	3 (8)	0.11
Luminex-DSA positive, N (%)	15 (20)	36 (95)	<0.001
Class I	4 (5)^b^	22 (58)	<0.001
Class II	9 (12)^c^	9 (24)	0.011
Both	2 (3)^d^	5 (13)	0.033
sCD30 positive, N (%)			
sCD30	31 (41)	17 (45)	0.84
sCD30 and DSA	0 (0)	16 (42)	<0.001
**Procedures and follow-up**			
Pre-tx immunoadsorption			
Patients, N (%)	0 (0)	38 (100)	<0.001
Treatments, median (range)	0 (0)	8 (4–22)	<0.001
Pre-tx plasma exchange			
Patients, N (%)	1 (1)	12 (32)	<0.001
Treatments, median (range)	0 (0–1)	0 (0–6)	<0.001
Post-tx immunoadsorption or plasma exchange			
Patients, N (%)	1 (1)	36 (95)	<0.001
Treatments, median (range)	0 (0–2)	4 (0–18)	<0.001
Induction therapy, N (%)			
No induction	7 (9)	0 (0)	0.093
Anti-CD20 rituximab	1 (1)	36 (95)	<0.001
Basiliximab	68 (89)	18 (47)	<0.001
Thymoglobulin	1 (1)	20 (53)	<0.001
Initial calcineurin inhibitor, N (%)			
Cyclosporine	55 (72)	1 (3)	<0.001
Tacrolimus	21 (28)	37 (97)	<0.001
Post-tx hospital stay (days), median (range)	13 (9–57)	18 (10–57)	<0.001
Follow-up (months), median (range)	51 (11–121)	43 (7–97)	0.20

* *Comorbidities at time of transplantation (diabetes and arterial hypertension with treatment indication, cardiovascular event defined as s/p stroke or PCI/CAB surgery)*.

a *Unspecific, most likely due to autoantibodies*.

b *Below 1,000 MFI*.

c *Below 1,000 MFI (N = 7), or considered unspecific/irrelevant (N = 2)*.

d *HLA antibodies were identified only retrospectively (before Luminex era)*. *ADPKD, autosomal-dominant polycystic kidney disease; DSA, donor-specific human leukocyte antigen (HLA) antibodies; ESRD, end-stage renal disease; HD, hemodialysis; N, number; PD, peritoneal dialysis; tx, transplant; XM, crossmatch*.

### Graft Survival and Function

**Figure 2:**
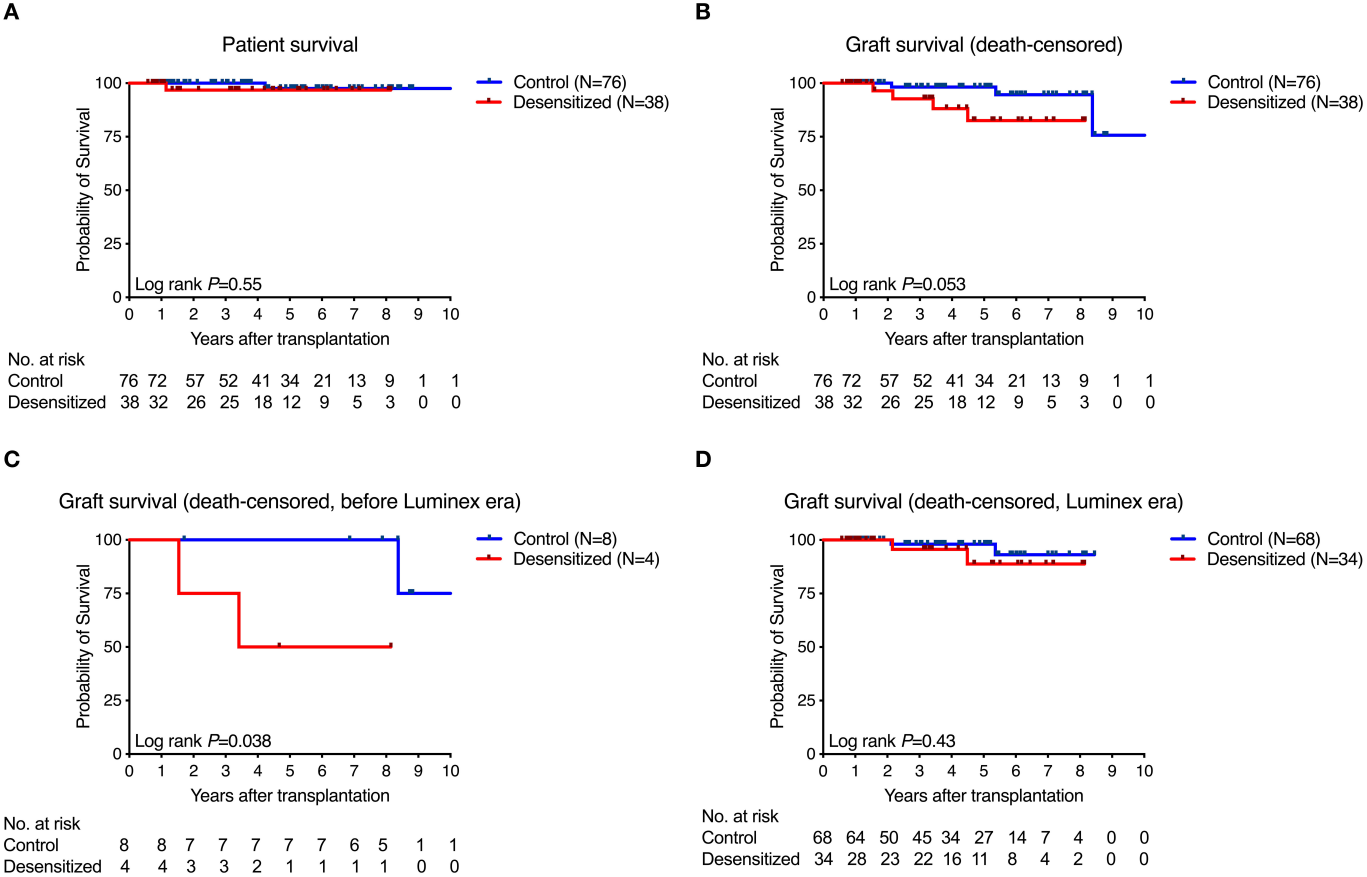
Patient and graft survival. Patient survival did not significantly differ between groups **(A)** while there was a trend toward reduced death-censored graft survival **(B)** that was driven by results obtained from the era before Luminex testing became available in clinical routine **(C)**. In contrast, death-censored graft survival in both groups was nearly identical in the Luminex era **(D)**.

Figure 2 shows patient and graft survival. Patient survival did not significantly differ between desensitized and standard-risk control patients (*P* = 0.55; Figure 2A). There was a trend toward reduced death-censored graft survival in desensitized patients (*P* = 0.053, Figure 2B) that was mainly driven by reduced survival in the early era before highly sensitive Luminex testing was introduced into clinical routine at our center (*P* = 0.038; Figure 2C). In the Luminex era (from March 2009), however, death-censored graft survival did not significantly differ between the two groups (*P* = 0.43; Figure 2D).

Serum creatinine, MDRD-GFR and protein-to-creatinine ratio was also not significantly different between the two groups. At day 360 after transplantation, median serum creatinine was 1.36 mg/dL in desensitized and 1.38 mg/dL in standard-risk patients (*P* = 0.88). The respective numbers for MDRD-GFR were 55 and 54 mL/min (*P* = 0.38), and for protein-to-creatinine-ratio 11 and 14 g/mol creatinine (*P* = 0.35).

### Rejection Episodes

The incidence of rejection episodes without borderline changes were with 21% not significantly different in desensitized patients from the 18% rate in standard-risk patients (*P* = 0.74, Table 2). AMR were more frequent in desensitized than in standard-risk recipients (16 vs. 7%, *P* = 0.12), without reaching statistical significance.

**Table 2:** Rejection and complications.

	**Standard risk (*N* = 76)**	**Desensitized** **(*N* = 38)**	***P* value**
**Allograft rejection (BANFF 2017)**			
At least one rejection episode (excluding Borderline changes), N (%)	14 (18)	8 (21)	0.74
TCMR, N (%)	9 (12)	3 (8)	0.75
TCMR IA	7 (9)	3 (8)	1.0
TCMR IB	2 (3)	0 (0)	0.56
TCMR II/III	0 (0)	0 (0)	1.0
AMR, N (%)	5 (7)	6 (16)	0.12
**Delayed graft function, N (%)^a^**	1 (1)	1 (3)	0.62
**Infectious complications**			
Viral, N (%)			
Polyoma virus replication^b^	3 (4)	4 (11)	0.24
BKVAN^c^	1 (1)	2 (5)	0.22
CMV^d^	13 (17)	10 (26)	0.18
Bacterial, N (%)			
Urosepsis	6 (8)	4 (11)	0.57
Pneumonia	11 (14)	8 (21)	0.44
Wound infection	3 (4)	1 (3)	0.70
CVC-associated infection	6 (8)	3 (8)	0.31
Fungal, N (%)	4 (5)	3 (8)	0.16
**Surgical complications**			
Lymphocele^e^, N (%)	14 (18)	7 (18)	0.12
Bleeding^f^, N (%)	11 (14)	15 (39)	0.004

a *Dialysis within the first week after transplantation, except single dialysis for hyperkalemia*.

b *>10,000 copies/mL*.

c *SV-40-positive*.

d *>1,000 copies/mL*.

e *Requiring intervention*.

f *Requiring intervention or blood transfusion, AMR: antibody-mediated rejection*. *BKVAN, BK virus-associated nephropathy; CMV, cytomegalovirus; CVC, central venous catheter; TCMR, T cell-mediated rejection; N, number*.

### Infectious and Surgical Complications

Table 2 summarizes the infectious and surgical complications. Viral infections, such as cytomegalovirus infection (*P* = 0.18) or polyoma virus replication (*P* = 0.24), tended to be more frequent in desensitized compared to standard-risk recipients. No significant differences were found for bacterial (*P* = 0.71) or fungal infections (*P* = 0.16). Surgical complications, such as lymphoceles, were also not significantly different between both groups (*P* = 0.12) while a higher frequency of bleeding complications was observed in desensitized patients (*P* = 0.004), most likely due to the perioperative desensitization therapy.

### Desensitization and HLA Antibodies

**Figure 3:**
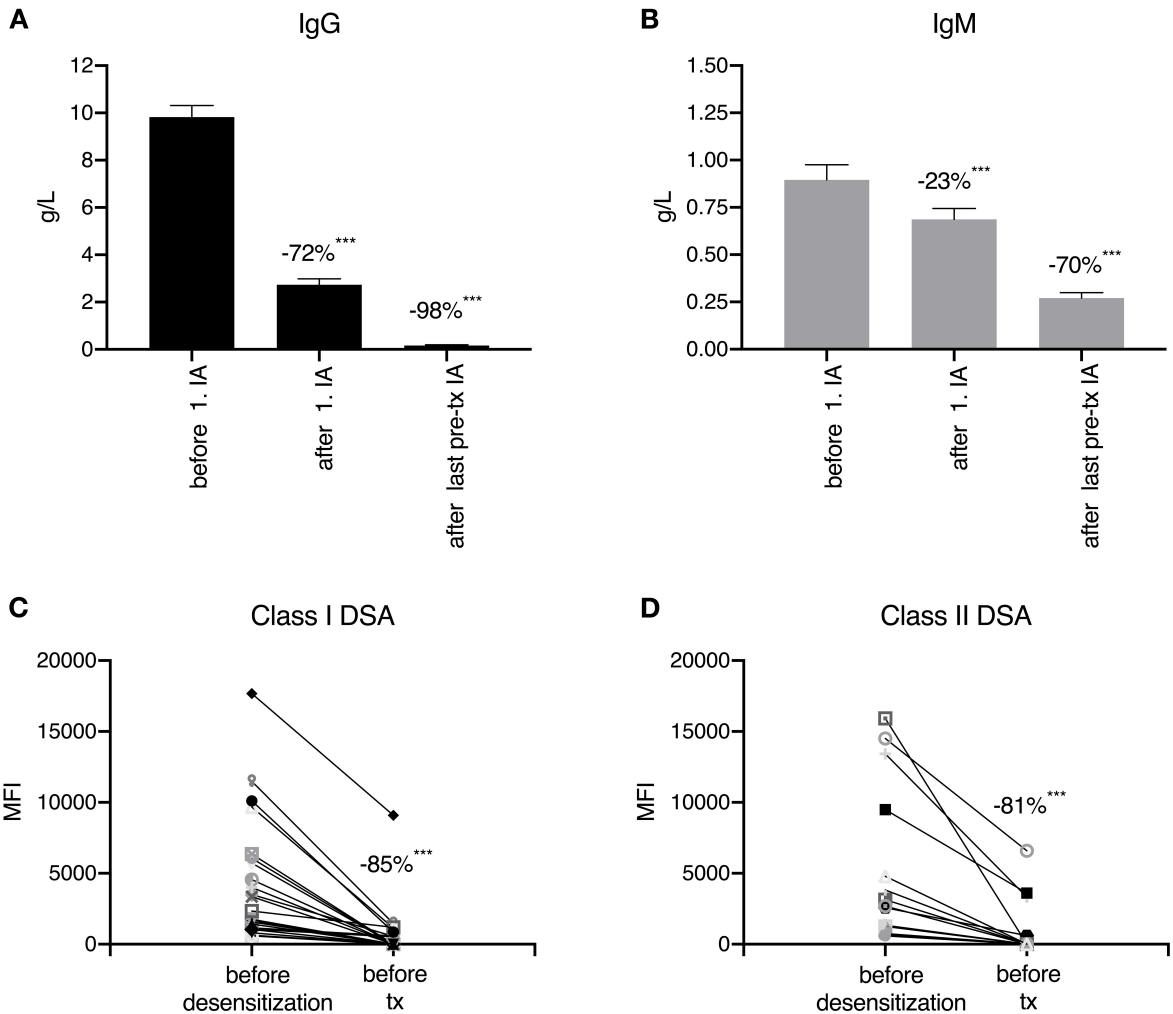
Immunoglobulin **(A,B)** and donor-specific human leukocyte antigen antibody **(C,D)** reduction during desensitization. After the first immunoadsorption (IA) session, total IgG was reduced by 72% **(A)** and total IgM by 23% **(B)**. After a median of 8 IA treatments, the reduction in IgG and IgM reached 98 and 70%, respectively. During pre-transplant desensitization (immunoadsorption and plasmapheresis), human leukocyte (HLA) class I donor-specific HLA antibodies (DSA) were reduced by 85% and HLA class II DSA by 81%. ***p < 0.001.

After a median of 8 pre-transplant IA treatments, total IgG was reduced by 98%, total IgM by 70%, HLA class I DSA by 85% and HLA class II DSA by 81% (*P* < 0.001 for all; Figure 3). After desensitization and before transplantation, all DSA were completely eliminated, according to the antibody detection technique at the respective time point. Table 3 summarizes the outcomes of desensitized patients according to their pre- and post-transplant presence of DSA. Two of 38 patients (number 22 and 23) had no DSA but a positive B cell crossmatch result. These patients showed primary graft function, no AMR, and no graft loss or death during clinical follow-up. Of 36 patients with pre-transplant DSA in the range of 500 to 999 MFI (*N* = 3) or 1,000 to 17,682 MFI (*N* = 33), 20 patients (56%) completely had lost their DSA on day 360 after transplantation. Only 1 of these 20 patients (6%) with a pre-transplant DSA of 2,698 MFI against HLA DRB1^*^03:01 (DR17) experienced AMR early after transplantation, and lost her graft more than 4 years after transplantation after cardiac surgery and infectious complications. Eleven patients showed persistence of DSA that had already been identified pre-transplant. Two of them (18%) suffered from AMR without graft loss and one patient lost her graft without signs of AMR. AMR was as high as 60% when the 5 patients were analyzed who had persistent DSA on day 360 together with *de novo* DSA development at some time during the first year after transplantation; two of these five patients lost their allograft. Compared to patients with loss of DSA, patients with persistent DSA had a higher MFI before desensitization (4,034, range 1,426–15,918 vs. 1,701, range 596–17,682, *P* < 0.001). Another two patients had (transient) *de novo* DSA without persistent DSA that disappeared during further follow-up with no AMR or graft loss.

**Table 3:** Donor-specific HLA antibody results and outcomes.

**Pat**.	**Before desensitization**	**After desensitization^a^**	**Day 30**	**Day 180**	**Day 360**	**Event**
1	B*07:02 (10,111)	B*07:02 (861)^b^	-	-	B*07:02 (740)	GL
	B*40:01 (B60) (7,282)	B*40:01(B60) (849)^b^	-	-	-	
2	DRB1*01:01 (3,996)	-	-	DRB1*01:01 (703)	DRB1*01:01 (1,037)	AMR
	DQB1*05:01 (3,048)	-	DQB1*05:01 (1,110)	DQB1*05:01 (1,033)	DQB1*05:01 (1,273)	GL
	DPB1*02:01 (9,486)	DPB1*02:01 (3,611)^b^	DPB1*02:01 (6,832)	DPB1*02:01 (8,625)	DPB1*02:01 (10,661)	
			DPA1*01:03 (6,832)	DPA1*01:03 (9,696)	DPA1*01:03 (11,387)	
3	A*03:01(1,432)	-	-	-	-	
	A*29:01 (2,919, IgM: 476)	-	-	-	-	
4	A*03:01 (813)	-	-	-	-	
5	B*44:03 (17,682)	B*44:03 (9,096)	B*44:03 (1,138)	B*44:03 (524)	-	
6	DQ7 (14,509)	DQ7 (6,588)^c^	DQ7 (13,363)	DQ7 (18,529)	DQ7 (11,938)	AMR, GL
	DQA1*05:05 (13,581)	DQA1*05:05 (6,557)^c^	DQA1*05:05 (13,349)	DQA1*05:05 (18,529)	DQA1*05:05 (2675)	
			A*32:01 (689)	A*32:01 (771)		
7	A*01:01 (2,335)	A*01:01 (1,199)	A*01:01 (4,040)	A*01:01 (5,736)	A*01:01 (2,109)	AMR
			DRB1*01:01 (2,717)	DRB1*01:01 (6,248)	DRB1*01:01 (1,792)	
				C*05:01 (3,368)	C*05:01 (600)	
				DQB1*05:01 (885)		
				DQB1*06:03 (636)		
				DQA1*01:03 (769)		
8	A*24:02 (1,304)	-	-	-	-	AMR
	B*18:01 (9,676)	B*18:01 (1,138)	B*18:01 (1,370)	B*18:01 (808)	B*18:01 (566)	
	B*37:01 (2,516)	-	-	-	-	
	DRB1*16:01 (4,798)	-	DRB1*16:01 (1,526)	DRB1*16:01 (937)	DRB1*16:01 (1,454)	
	DRB5*01:01 (DR51) (1,653)	-	-	-	-	
	DQB1*06:02 (1,156)	-	-	-	-	
9	C*12:03 (IgM: 1,760)	-	-	-	-	
10	A*02:01 (1,050)	-	-	-	-	
11	DRB1*13:01 (IgM: 686)	-	DRB1*13:01 (IgM: 679)	-	-	
12	A*01:01 (1,579)	-	-	A*01:01 (1,010)	A*01:01 (779)	AMR
13	B*18:01 (4,012)	-	-	-	-	
	DRB1*03:01 (DR17) (553)	-	-	-	-	
	DQB1*02:01 (13,433)	-	-	-	-	
	DQA1*05:01 (13,433)	DQA1*05:01 (3,319)	DQA1*05:01 (898)	-	-	
14	A*02:01 (3,355)	-	A*02:01 (1,268)	-	-	
15	DRB1*03:01(DR17) (2698)	-	-	-	-	AMR, GL
16	DQB1*02:01 (15,918)	-	DQB1*02:01 (9,756)	DQB1*02:01 (13,107)	DQB1*02:01 (13,583)	
	DQA1*05:01 (14,587)	DQA1*05:01 (2,135)	DQA1*05:01 (6,822)	DQA1*05:01 (12,174)	DQA1*05:01 (12,729)	
17	C*07:02 (IgM: 596)	-	-	-	-	
18	A*02:01 (6,355)	-	-	-	-	
	A*69:01 (3,191)	-	-	-	-	
	B*44:02 (3,956)	-	-	-	-	
		-	-	-	C*05:01 (5,875)	
19	DRB1*03:01(DR17) (2,568)	DRB1*03:01(DR17) (600)	DRB1*03:01(DR17) (878)	-	DRB1*03:01(DR17) (1,277)	
20	B*51:01 (1,717)	-	-	-	-	
	DRB1*07:01 (752)	-	-	-	-	
21	A*01:01 (11,485)	A*01:01 (1,464)	A*01:01 (6,865)	-	A*01:01 (5,253)	
22		-	-	-	-	
23		-	-	-	-	
24	DRB1*13:01 (3,828)	-	-	-	-	
	DRB3*01:01(DR52) (776)	-	-	-	-	
25	A*23:01 (1,426)	-	A*23:01 (568)	-	A*23:01 (1,464)	
			C*17:01 (591)			
			DRB1*07:01 (515)			
26	A*24:02 (2,891)	-	A*24:02 (511)	-	-	
	C*12:03 (6,064)	-	C*12:03 (512)	-	-	
	DPB1*15:01 (588)	-	DPB1*15:01 (600)	-	-	
27	A*01:01 (1,684)	-	-	-	-	
28	C*05:01 (1,683)	-	C*05:01 (570)	-	C*05:01 (582)	Death
29	A*32:01 (4,562)	-	A*32:01 (3,597)	-	A*32:01 (5,774)	
			B*08:01 (680)			
30	A*26:01 (IgM: 822)	-	-	-	-	
	B*13:02 (IgM: 1,466)	-	-	-	-	
	C*02:02 (IgM: 520)	-	-	-	-	
	C*06:02 (IgM: 1,591)	-	-	-	-	
31	B*58:01 (645)	-	-	-	-	
	DQB1*06:09 (1,266)	-	-	-	-	
	DQA1*01:02 (1,266)	-	-	-	-	
32	DQA1*03:01 (3,139)	-	-	DQA1*03:01 (2,040)	DQA1*03:01 (1,759)	
	DQ8 (2,539)	-	-	-	-	
33	DQB1*06:03 (1,339)	-	-	-	-	
	DQA1*01:03 (1,339)	-	-	-	-	
34	A*02:01 (IgM: 1,061)	A*02:01 (IgM: 529)	-	-	-	
	C*04:01 (IgM: 562)	-	-	-	-	
35	C*14:02 (5,728)	-	C*14:02 (587)	-	C*14:02 (1,717)	
36	A*02:01 (IgM: 1,164)	A*02:01 (IgM: 597)	-	-	-	
		C*03:04(Cw10) (IgM: 705)				
		DQB1*05:01 (IgM: 865)				
37	B*51:01 (IgM: 559)	-	-	-	-	
	C*04:01 (IgM: 1,642)	-	-	-	C*04:01 (755)	
38	B*73:01 (3,505)	B*73:01 (566)	B*73:01 (1,139)	B*73:01 (4,028)	B*73:01 (3,180)	
	76–100% reduction					
	51–75% reduction					
	26–50% reduction					
	25% reduction to 25% increase					
	26–50% increase					
	51–75% increase					
	>75% increase					
	*de novo* DSA					

a *After desensitization, some of the donor-specific HLA antibodies lie above the predefined threshold of 1.000 MFI. Unless otherwise indicated (footnotes b and c), these antibodies were identified only retrospectively during reanalysis when EDTA inactivation was replaced by heat inactivation*.

b *Before routine Luminex testing, antibodies were identified retrospectively*.

c *Before routine donor typing for HLA-C, -DQ, -DP locus antigens, antibodies were identified retrospectively*. *AMR, antibody-mediated rejection; DSA, donor-specific human leukocyte antigen (HLA) antibodies; GL, graft loss*.

Sixteen patients (44%) with pre-transplant DSA also had an sCD30 value of ≥80 ng/mL prior to transplantation as an indication of a pre-activated immune system. Nine of these 16 patients (56%) had persistent DSA and 4/16 patients (25%) suffered from AMR compared to only 7/20 (35%, *P* = 0.31) and 2/20 patients (10%, *P* = 0.34), respectively, with an sCD30 value below 80 ng/mL (*P* = 0.38). Most importantly, graft loss in patients with pre-transplant DSA was observed in 4/16 patients (25%) who were sCD30 positive, while only 1/20 patients (5%) with an sCD30 value below the cut-off experienced graft loss (*P* = 0.15) translating in a sensitivity of 80% and a NPV of 95%.

## Discussion

Several desensitization strategies have been published that allow transplantation across the HLA-antibody barrier. Most published protocols have used plasmapheresis and intravenous immunoglobulins, with graft survival rates ranging from 77% to 94% depending on the degree of sensitization, and concomitant AMR rates up to 15% (8, 11–15). Our group has developed a strategy to eliminate preexisting DSA by immunoadsorption, allowing safe transplantation even in highly sensitized recipients. Thorough pre-transplant risk stratification and effective antibody elimination combined with post-transplant antibody monitoring reduced AMR rates and improved graft survival. We present here the results of 38 desensitized patients transplanted at our center from 2007 to 2016 and compare them with 76 standard-risk patients who were matched for time after transplantation.

Desensitized patients had patient and graft survival rates that were not significantly different from those of standard-risk recipients, whereas desensitized patients showed a trend toward lower death-censored graft survival (*P* = 0.053). However, this trend disappeared when the 34 patients transplanted after the introduction of the sensitive Luminex-SAB assay were analyzed. The Luminex-SAB procedure, which allows more sensitive detection of DSA, was introduced into pre-transplant identification as part of the “Heidelberg algorithm” starting in April 2009 (9). An MFI value above 1,000 was classified as a risk factor for immunologic graft loss. More recently, our group has introduced another biomarker to further improve pre-transplant risk assessment. Since 2016, the immune activation marker sCD30 has been integrated into our algorithm to identify patients at high immunological risk before transplantation. Several studies in deceased donor transplant recipients have shown that determination of sCD30 with a cut-off value of 80 ng/mL before transplantation is beneficial, as patients with the co-presence of DSA (MFI ≥ 1,000) and sCD30 (≥80 ng/mL) before transplantation were shown to be at a significantly higher risk for AMR and graft loss than patients with DSA but without sCD30 positivity (10, 16). These patients may require a more intensive induction regimen including thymoglobulin as well as special care after renal transplantation. The present study in living donor transplant recipients shows a similar trend as in previous studies. Desensitized patients with both DSA- and sCD30-positivity before transplantation were at a higher risk for graft loss compared to patients without sCD30 positivity, however, without reaching statistical significance.

Living kidney transplant recipients were treated with repeated IA until the CDC crossmatch became negative and measured DSA were below the 1,000 MFI threshold. A median of 8 IA treatments before transplantation reduced IgG by 98% and IgM by 78% in sensitized patients. IA can treat large plasma volumes, which may improve antibody removal compared with desensitization with plasmapheresis: 95% of all patients studied were successfully desensitized and transplanted, compared with the much lower frequency of only 80% reported when plasmapheresis and intravenous immunoglobulins were used (17). Another advantage of IA is its specificity: bleeding complications in terms of transfusion requirement or intervention occur at a higher rate of up to 70% with multiple plasmapheresis treatments than with our desensitization protocol (17). Compared with newer desensitization strategies such as desensitization with imlifidase, immunoadsorption allows better risk assessment before transplantation. DSA that can be easily removed by IA before transplantation are more likely to be lost after transplantation. Conversely, antibodies that cannot be removed by IA may persist after transplantation and damage the renal graft (18). In contrast, imlifidase degrades IgG, and DSA are lost for several days regardless of the level of re-synthesis, making risk assessment impossible (19). Another advantage of IA compared to imlifidase is the fact that it can be used several times and even for weeks or months, while imlifidase may be given only once (20).

In previous studies, graft survival at two years was only about 50% when patients were desensitized prior to transplantation (21). Importantly, when IA is used as the main method of desensitization, antibody rebound after treatment must be considered. Therefore, according to our protocol, we performed repeated IA after transplantation in addition to the immunosuppression described above until good graft function was achieved (e.g., serum creatinine < 2.0 mg/dl). Interestingly, DSA remained below a threshold of 1,000 MFI in 6 of 10 sensitized patients during the post-transplant observation period in a previously published cohort (5). In the present study, 20 of 36 patients (56%) had persistently negative DSA on day 360 after transplantation.

The use of strong immunosuppression may lead to negative side effects such as infectious complications. However, in the present study, no significant difference was observed, neither with respect to bacterial infections nor with respect to viral infections such as cytomegalovirus or polyoma infections. Thus, the present study demonstrates that treatment with repeated IA is feasible without severe infectious complications and without the need to replace immunoglobulins (22). Since April 2009, two depleting antibodies, thymoglobulin and rituximab, have been used in combination. In the present study, there is no evidence of increased infectious complications, but probably the numbers are too small. In the field of ABO-incompatible renal transplantation, more infectious complications have been observed probably due to the use of rituximab (23). Therefore, studies analyzing possible infectious side effects in sensitized renal transplant recipients are needed.

Limitations of the present study include the retrospective and monocentric character of the study with a rather small number of patients who experienced only a limited number of adverse events such as AMR and graft loss due to AMR as well as differences in the immunosuppressive regimens between sensitized (mostly tacrolimus as calcineurin-inhibitor) and standard risk (mostly cyclosporine A as calcineurin-inhibitor) patients.

In conclusion, both pre-transplant characterization of the immune status of sensitized kidney transplant recipients by measuring B-cell (DSA) and T-cell (sCD30) activity and post-transplant monitoring (DSA and non-DSA) play an important role in the management of living kidney transplantation across the HLA barrier. The algorithm we have described for identifying sensitized living kidney transplant recipients, consistent desensitization with repeated IA, and the peri-graft management described lead to good graft outcomes with side effects comparable to those of standard-risk recipients.

## Data Availability Statement

The original contributions presented in the study are included in the article/Supplementary Material, further inquiries can be directed to the corresponding author/s.

## Author Contributions

FK, CSü, and CM designed the study. FK and LPdS performed the study. CSp, LB, CN, MS, JB, LP, HT, CSo, AM, and MZ contributed important patients. FK, CSü, LPdS, and CM analyzed the data and wrote the manuscript. All authors contributed to the final version of the manuscript and approved it.

## Funding

This study was funded in part by the Dietmar-Hopp Stiftung.

## Conflict of Interest

The authors declare that the research was conducted in the absence of any commercial or financial relationships that could be construed as a potential conflict of interest.

## Publisher's Note

All claims expressed in this article are solely those of the authors and do not necessarily represent those of their affiliated organizations, or those of the publisher, the editors and the reviewers. Any product that may be evaluated in this article, or claim that may be made by its manufacturer, is not guaranteed or endorsed by the publisher.
