# Supplementary material for: Living Donor Kidney Transplantation in Patients With Donor-Specific HLA Antibodies After Desensitization With Immunoadsorption
Source: Front Med (Lausanne). 2021 Dec 17;8:781491. doi: 10.3389/fmed.2021.781491 (PMC8719417; doi:10.3389/fmed.2021.781491)
Supplement: Supplementary file 1 [file Table_1.docx]

**Supplemental Table 1: “Heidelberg Algorithm” (applied since April 2006) (**[**7-9**](file:///N:\Kaelble_Living%20donation%20and%20HLA%20antibodies_revised%20file_tracked%20%20%20changes.docx#_ENREF_7)**)**

| **(1) Pretransplant identification of high-risk patients**  *Donor-independent*  (i) CDC-PRA-DTT ≥85% (current or historical)  (ii) HLA class I and II antibody positivity in ELISA^*^  (iii) HLA class I positivity in ELISA* (retransplant)  *Donor-dependent*  (i) Positive CDC B-cell crossmatch with HLA class II antibody positivity in ELISA^*^ (retransplant)  (ii) Positive CDC T-cell crossmatch  (iii) DSA ≥1,000 MFI (living donor; since April 2009)  (iv) DSA ≥1,000 MFI and sCD30 ≥80 ng/mL (since October 2016) |
| --- |
| **(2) Good HLA match in patients with HLA class I and class II antibody positivity in ELISA^*^ (deceased donor)**  (i) CDC-PRA-DTT ≥10%: 0−1 HLA-A+B+DR mismatches  (ii) CDC-PRA-DTT <10%: 0−2 HLA-A+B+DR mismatches |
| **(3) Acceptable Mismatch Program of Eurotransplant (deceased donor)**  (i) CDC-PRA-DTT ≥85% (current or historical) |
| **(4) Pretransplant treatment**  (i) Single plasmapheresis (deceased donor)  (ii) Repeated immunoadsorption (living donor)  (iii) Triple immunosuppression (tacrolimus + enteric-coated mycophenolic sodium + methylprednisolone; in the case of living donor, together with the initiation of apheresis therapy)  (iv) Rituximab 375 mg/m2 (when all crossmatches are negative)  (v) Thymoglobulin 1.5 mg/kg body weight preoperatively and a median of 2 times (range: 1–6) postoperatively (since April 2009; IL-2 receptor antagonist basiliximab before April 2009^#^) |
| **(5) Posttransplant treatment**  (i) Repeated plasmapheresis (deceased donor)  (ii) Repeated immunoadsorption (living donor) |
| **(6) Protocol biopsies**  (i) On days 7 and 90 (since November 2007) |
| **(7) Posttransplant monitoring of DSA**  (i) On days 0, 7, 30, 180, and every 6 months thereafter  (ii) If deterioration of allograft function  (iii) C1q assay if DSA ≥3,000 MFI (since March 2016) |

Adopted from ([7-9](file:///N:\Kaelble_Living%20donation%20and%20HLA%20antibodies_revised%20file_tracked%20%20%20changes.docx#_ENREF_7)). CDC: complement-dependent cytotoxicity, PRA: panel reactive antibodies, DTT: dithiothreitol, DSA: donor-specific HLA antibodies, sCD30: soluble CD30. *more recently, ELISA screening was substituted by Luminex screening, ^#^basiliximab (20 mg on days 0 and 4 after transplantation) continued to be given in those patients who possessed low-level DSA that were identified in Luminex testing only but who had a negative crossmatch result (and a sCD30 concentration below 80 ng/mL, from October 2016).
